# Exhale-Dx™: A non-invasive, real-time breath analysis system using deep learning for asthma diagnosis

**DOI:** 10.14440/jbm.2024.0142

**Published:** 2025-07-11

**Authors:** Hanya Ahmed, Jona Angelica Flavier, Victor Higgs

**Affiliations:** Department of Research and Development, Applied Nanodetectors Ltd., London N5 2EF, United Kingdom

**Keywords:** Asthma, Volatile organic compounds, Diagnosis, Deep neural networks

## Abstract

**Background:**

Asthma presents significant diagnostic and therapeutic challenges, impacting millions and posing a substantial burden on healthcare systems, particularly in the United Kingdom, where it afflicts roughly 5.4 million individuals. Severe asthma, incurring over 50% of total expenditures, tends to lead to frequent exacerbations and preventable emergency admissions. Traditional diagnostic methods, primarily based on clinical history, can result in delays and misdiagnoses, culpable for over 1,200 deaths annually, 90% of which are considered preventable with timely intervention.

**Objective:**

To address this issue, we developed Exhale-Dx™, a point-of-care breath test platform that provides a non-invasive, user-friendly solution for asthma diagnosis and monitoring. Exhale-Dx™ captures volatile organic compounds (VOCs) in exhaled breath, reflecting real-time metabolic and inflammatory markers of lung function. By analyzing these personalized breath signatures, clinicians and patients can detect exacerbations up to three days in advance, thus facilitating early and targeted interventions to reduce emergency care utilization. The system integrates capnographic waveforms, asthma control scores, and clinical lung function data, offering a comprehensive diagnostic profile.

**Methods:**

Using Exhale-Dx™ data, we developed the Asthma Diagnostic Enhanced Neural Architecture (ADENA), an advanced deep neural network that leverages VOC biomarkers and lung function data to enhance diagnostic precision.

**Results:**

ADENA achieved exceptional performance, delivering 98.7% accuracy, an F1 score of 0.98, and a low mean squared error of 0.065. The deconvolution analysis further confirmed the model’s ability to detect significant physiological differences between asthmatic and non-asthmatic profiles.

**Conclusion:**

Our findings showed that VOC analysis combined with advanced neural networks could accurately distinguish asthmatic profiles, highlighting their potential for early, non-invasive interventions in respiratory health diagnostics.

## 1. Introduction

Asthma, a chronic respiratory condition affecting approximately 262 million people globally, continues to pose significant challenges in diagnosis and management.^1^ Conventionally, diagnosing asthma has relied heavily on clinical history, an approach that, while essential, often falls short of ensuring timely and accurate identification of the condition. Misdiagnosis or delayed diagnosis not only compromises patient care but also exacerbates the disease’s impact on health systems and societies across the globe. In the United Kingdom alone, asthma affects 5.4 million individuals and poses an annual economic burden of over £1.1 billion on the National Health Service.^2^ This includes costs from hospital admissions, prescription medications, and millions of general practitioner visits. Despite these substantial investments, preventable outcomes linger, with virtually 65,000 emergency asthma admissions and over 1,200 avoidable deaths reported annually.^3^ Severe asthma, which is culpable for more than half of asthma-related healthcare costs, adds to this strain, emphasizing the critical need for improved diagnostic and monitoring methods.

The challenges of asthma diagnosis are particularly pronounced in children, in whom it remains the most common chronic respiratory disease, affecting 11.9 million in the European Union alone.^4^ In pediatric cases, reliance on clinical history often leads to misdiagnosis, either through overdiagnosis or underdiagnosis. Symptoms common to other respiratory conditions, such as viral infections, further complicate accurate identification. Overdiagnosis can result in unnecessary corticosteroid treatments, raising healthcare costs and exposing children to unwarranted side effects. Conversely, under-diagnosis leaves children vulnerable to unnecessary morbidity, poor quality of life, and increased mortality, especially in low-resource settings. Compounding the issue, traditional lung function tests are often too complex for young children, rendering clinicians without objective, child-friendly diagnostic tools. This underscores an urgent demand for accessible, non-invasive testing methods that can reliably distinguish asthma from other conditions.

Emerging technologies offer a beacon of hope in addressing these gaps. Advances in non-invasive diagnostic tools, such as breath analysis and carbon dioxide (CO_2_) capnographic data, provide innovative pathways for asthma diagnosis and monitoring.^5^ Volatile organic compounds (VOCs) in exhaled breath have been shown to correlate with airway inflammation, offering a real-time, personalized diagnostic approach.^6^ Similarly, CO_2_ capnograms offer distinct patterns – healthy individuals exhibit square-shaped waveforms, while asthma patients show characteristic “shark-fin” waveforms – allowing for clear differentiation between asthmatic and non-asthmatic conditions.^7^ These tools are promising not only for accurate diagnosis but also for monitoring exacerbations, as deviations in waveform properties, such as angles and plateaus, can signify worsening conditions. Fractional exhaled nitric oxide (FeNO) is a well-established, non-invasive biomarker for assessing type 2 (T2) airway inflammation in asthma and is commonly used in clinical practice to support diagnosis and guide treatment decisions, particularly in cases of eosinophilic or steroid-responsive asthma. While the Exhale-Dx™ platform focuses on breath-based VOC profiling and capnographic analysis, it does not currently include FeNO measurement.

Other non-invasive measures of airway inflammation observed in asthmatic individuals include distinct physiological characteristics, such as higher airway temperature and altered humidity levels, compared to their non-asthmatic counterparts. This temperature elevation is linked to inflammatory processes, which generate heat as a byproduct of cellular activity in the airways. Furthermore, in more severe cases of asthma, where inflammation is more pronounced, the disparity in temperature becomes even more significant and is accompanied by a reduction in humidity.^8^ The decreased humidity can be attributed to airway narrowing and impaired mucosal hydration caused by inflammation, which hinders the natural humidification process during exhalation.

In addition to temperature and humidity, other non-invasive parameters, such as pressure and airflow, play a vital role in characterizing airway function in asthmatics.^9^ Studies have shown that individuals with asthma experience reduced airflow rates, particularly during expiration, due to the narrowing and obstruction of airways caused by bronchoconstriction and mucus buildup. This reduced airflow, measured as peak expiratory flow rate or forced expiratory volume in 1 s, provides a clear indication of airway restriction. Similarly, alterations in airway pressure dynamics can reflect the increased resistance encountered during breathing in asthmatic patients. These changes in pressure and flow are significant markers that help differentiate asthma severity levels and monitor disease progression. By leveraging these non-invasive biomarkers, clinicians can gain valuable insights into the underlying inflammatory and mechanical abnormalities in asthma.

The integration of advanced machine learning techniques has further revolutionized asthma diagnostics. Traditional models, such as support vector machines (SVMs) and random forests initially demonstrated potential but were often limited by their dependence on manual feature extraction and their inability to fully capture the complex, non-linear relationships inherent in patient data.^8^,^10^ Recently, deep neural networks have emerged as powerful tools for processing large, labeled datasets and identifying intricate patterns in clinical and demographic data. These models have shown impressive accuracy in asthma diagnosis; however, challenges remain, particularly regarding the need for extensive, high-quality datasets and their susceptibility to data variability in real-world clinical settings.^11^,^12^

This study investigated a novel diagnostic framework, the Asthma Diagnostic Enhanced Neural Architecture (ADENA), designed to address limitations in present non-invasive asthma detection methods. The primary aim was to evaluate the ability of ADENA to process lung function and exhaled VOC data using deep learning algorithms for accurate asthma classification. The architecture incorporates an unsupervised learning component for feature extraction and is designed to function with limited and heterogeneous datasets, improving its applicability in clinical environments. By systematically evaluating model parameters, this research aimed to determine the diagnostic accuracy and computational efficiency of ADENA in both adult and pediatric populations.

In summary, the contributions of this paper are as follows: (i) A robust software algorithm is developed to reliably detect end-tidal plateaus, enabling reproducible and consistent biomarker measurements; (ii) By employing deconvolution analysis, ADENA provides nuanced insights into physiological differences between asthmatic and non-asthmatic profiles, uncovering critical diagnostic patterns; (iii) Through residual attention (RA) blocks and dynamic routing layers, the ADENA framework demonstrates superior diagnostic precision compared to traditional models. (iv) ADENA is adaptable for pediatric use, demonstrating its versatility and applicability across diverse patient demographics.

## 2. Methodology

### 2.1. Data pre-processing

Exhale-Dx™ leverages the sophisticated sensor array platform developed by Applied Nanodetectors Ltd and seamlessly integrates advanced nanotechnology with standard semiconductor processes. This integration enables high-volume production while maintaining the sensors’ high sensitivity and low power consumption. The sensors serve as both chemical biosensors and gas sensors, making them particularly suitable for real-time monitoring applications. Recognizing the critical need for standardized techniques in VOC breath analysis, Exhale-Dx™ incorporates an innovative end-tidal collection method that ensures the precise capture of breath samples from alveolar air. By monitoring physical parameters and regulating breath sample collection in real-time with fixed, controllable flow rates, the system guarantees accurate, consistent, and repeatable measurements.

The Exhale-Dx™ platform records 13 distinct waveforms during exhalation, capturing a wide range of data, including various VOCs (such as isoprene, acetone, and ethanol), CO_2_ parameters, peak exhaled flow rate, and additional physical variables, such as temperature and humidity. These comprehensive data streams give valuable insights into respiratory health and facilitate precise asthma diagnostics.

The Exhale-Dx™ device was rigorously tested on diverse age groups, including children aged six to 16 and adults aged 16 and older, with independent evaluations validating its accuracy and efficacy across physiological differences between age groups.^9^,^13^ In each case, the patient performed the test three times within 5 – 10 min. This validation underscores the device’s reliability as a trustworthy tool for asthma diagnostics, demonstrating its applicability to users of all ages.

**Figure 1:**
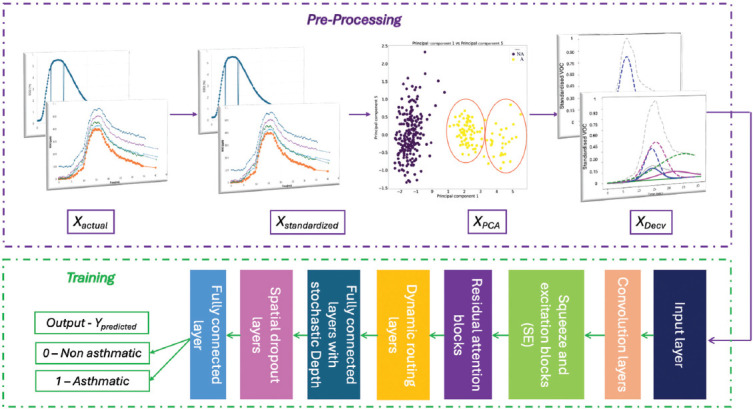
The architecture of the proposed framework for classifying asthmatic and non-asthmatic individuals using 13 different breath parameters, including volatile organic compounds. Data preprocessing includes standardization, principal component analysis, and deconvolution (Decv). The processed data were then fed into the Asthma Diagnostic Enhanced Neural Architecture for training, guided by the proposed loss function.

The diagnostic framework employed by Exhale-Dx™ is illustrated in Figure 1. Principal component analysis (PCA) acted as a pre-processing step, reducing the dimensionality of the input space while preserving the most critical features relevant to asthma diagnosis. By removing noise and redundancies, PCA uncovered underlying patterns in high-dimensional data, highlighting the VOCs that were most significant for diagnostic purposes. Further analysis through deconvolution quantified the differences between asthmatic and non-asthmatic profiles, providing additional diagnostic insights.

Following this pre-processing stage, the ADENA model was applied. ADENA incorporates two advanced neural network components: Squeeze-and-excitation modules and RA blocks. These components enhance feature representation, allowing the model to focus on clinically relevant data and improve diagnostic accuracy. By integrating these advanced neural blocks with unsupervised learning techniques, ADENA offers accurate, real-time asthma diagnosis even in the presence of sparse, noisy, or incomplete datasets.

This comprehensive framework, which combines Exhale-Dx™’s cutting-edge sensor technology with advanced machine learning models, delivers a transformative approach to asthma diagnostics. It effectively differentiated asthmatic profiles, providing reliable and actionable insights that pave the way for improved asthma diagnosis and patient care.

### 2.2. Principal component analysis and deconvolution

Before being incorporated into the diagnostic model, the 13 distinct waveforms underwent a standardization process to ensure uniformity and consistency throughout the dataset. This step was critical to the elimination of potential variability and discrepancies arising from differences in sampling conditions or measurement techniques. Once standardized, the waveforms were subjected to PCA, an essential pre-processing technique employed as part of an exploratory analysis. PCA served a dual purpose: reducing the dimensionality of the dataset and aiding in the optimization of hyperparameters for the selected deep learning algorithm. By distilling the waveforms into their most informative components, PCA not only enhanced computational efficiency but also provided a clearer understanding of the underlying data structure. Through dimensionality reduction, PCA isolated the most critical components in the data, assigning them higher weights in the model. These components represented the features most strongly associated with asthma diagnosis, ensuring the model being focused on the parameters that contribute most to diagnostic accuracy.

Following PCA, an additional layer of processing was applied through the deconvolution of breath biomarker signals. This step involved separating and quantifying each VOC present in the breath samples, allowing for a more detailed characterization of the biochemical markers associated with asthma. By deconstructing the composite signals into their individual components, deconvolution offered a more granular view of the biochemical processes underlying asthma. This enhanced the model’s ability to discriminate between asthmatic and non-asthmatic profiles, as well as between varying levels of asthma severity. This comprehensive pre-processing framework ensured that the model leveraged the most relevant features and biochemical signals, resulting in a more robust and accurate tool for asthma diagnosis.

### 2.3. Asthma diagnostic enhanced neural architecture

Principal component analysis functioned as a foundational component in the early layers of ADENA. It was used to analyze lung function parameters and biomarkers, assigning optimal weightings to the most informative features. By reducing the dimensionality of the input data, PCA eliminated noise and redundancies, ensuring that the model focused on critical patterns that differentiate asthmatic and non-asthmatic cases. This pre-processing stage enhanced the overall diagnostic accuracy and helped tune hyperparameters in the deep learning algorithm. The binary classification task in ADENA categorized profiles into two groups: asthmatic (1) and non-asthmatic (0).

The architecture began with an input layer that processed raw data from diverse biomarkers and lung function parameters. Convolutional layers follow, automatically detecting significant features and identifying patterns indicative of asthma. To further refine these patterns, squeeze-and-excitation blocks dynamically recalibrated feature maps, prioritizing the most relevant biomarkers. These blocks amplified the model’s ability to focus on informative channels, improving its precision in distinguishing asthma-specific characteristics. In addition, RA blocks enhanced the model’s sensitivity to subtle variations in lung function linked to asthma. By selectively attending to critical input regions, these blocks allowed the network to capture intricate patterns that might otherwise be overlooked.

*X′ =* σ(*w* * *X*+*b*) (I)


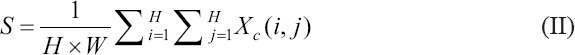


*E*=σ(W_2_δ(W_1_z_c_)) (III)

*RA* = *M*(*E*).*E* (IV)

output = *SAP* (*SD*(*DR*(*RA*))) (V)

In these equations, *X* is the input, *X’* is the convolution output, *w* is the convolution filter (weight), (weight), *b* is the bias term, *σ* represents the rectified linear unit function, *S* is the squeezed feature for the channel, *Xc*(*i*,*j*) is the input feature map at position (*i*,*j*), and *H* and *W* represent the height and width of the feature map. E is the excitation output, where *W_1_* and *W_2_* are weight matrices, and *δ* is the sigmoid activation function. The excitation was then fed into the RA blocks, where M(E) represents the implementation of the attention mechanism.

Following this, the output of the RA blocks was fed into dynamic routing layers, modeled after capsule networks.^14^ These layers improve contextual interpretation by preserving spatial hierarchies within the data. These layers permit the model to more accurately distinguish between asthmatic and non-asthmatic profiles by assisting it in identifying intricate patterns and relationships that conventional convolutional neural networks might overlook. Using stochastic depth for random layer skipping during training, the network employed fully-connected layers for final classification after the dynamic routing layers. This technique encourages the model to learn diverse feature representations. To further enhance generalization, spatial dropout layers were applied, randomly setting all feature maps to zero to prevent overfitting.^15^ Ultimately, a probability score (ranging from 0 to 1) was generated by a fully-connected layer using a sigmoid activation function, yielding the binary classification output: Asthmatic or non-asthmatic.

To train the ADENA framework, a comprehensive dataset comprising medical records, historical data, and clinician inputs was utilized. The model employed Adaptive Moment Estimation optimization algorithms to dynamically adjust the learning rate, ensuring efficient convergence during training. By incorporating advanced neural network components and optimization techniques, ADENA effectively identifies intricate patterns within the data, enabling precise asthma diagnosis.

The training process utilized a robust loss function designed to minimize discrepancies between predicted and actual outcomes. This custom loss function combined binary cross-entropy loss and focal loss to maximize performance. The focal loss component addressed class imbalances by reducing the weight of easily classified cases while emphasizing more complex ones, such as borderline asthmatic profiles. This allowed the model to focus on challenging cases, improving classification accuracy. Meanwhile, the binary cross-entropy loss component calculated the probability of predictions within a 0 – 1 range, penalizing misclassifications to refine the model’s sensitivity and accuracy.

*L_Loss_*= *L_focal_* + *L_BCE _*(VI)

*L_focal_*=−(1−*p_t_*)^γ^ *log*(*p_t_*) (VII)


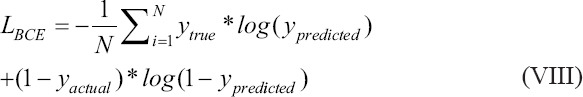


where, *y_actual_* is the actual data, *y_predicted_* is the predicted value, *N* is the number of data points, *p_t_* is the predicted probability, and *γ* is the focusing parameter.

To validate the model’s reliability and ensure its generalizability, cross-validation techniques were applied during the initial development phase. Despite a relatively small initial dataset, the data were divided into subsets for iterative training and validation. This five-fold cross-validation approach lowered the risk of overfitting, where the model became overly specialized to the training data, by evaluating performance on unseen data points. This process not only enhanced the model’s robustness but also provided valuable insights into its limitations, guiding future data collection efforts to support more comprehensive validation.

Extensive testing of the network was conducted under various configurations to identify optimal hyperparameters. Experiments included different learning rates (5.0 × 10^−3^, 1.0 × 10^−3^, 1.0 × 10^−4^, 1.0 × 10^−1^, 1.0 × 10^−2^), epochs (200, 500, and 1000), batch sizes (2 and 4), and training dataset sizes. These tests were implemented using TensorFlow and Keras frameworks, and all models were trained on an NVIDIA P100 graphics processing unit with 24 GB of memory. This rigorous evaluation ensured the model’s adaptability and high performance, even under varying conditions.

## 3. Results

### 3.1. Datasets

The study cohort consisted of 45 patients, comprising 25 women and 20 men, aged between 17 and 85 years. Of these, 20 individuals were asthmatic, actively managing their condition with medication prescribed following a diagnosis by their general practitioners. The remaining 25 participants served as a control group, with no known lung conditions or history of asthma. In addition, 20 younger patients aged six to 16 were included in the study, evenly split between 10 asthmatic patients and 10 individuals without any known lung conditions. Furthermore, 20 additional patients aged 17 and older were tested in a blind study, including 11 individuals with asthma and nine without any known lung conditions.

The dataset was divided into three portions for model development: 60% for training, 30% for validation, and 10% for testing. This random allocation ensured a balanced approach to model evaluation and reduced the risk of bias. During the training phase, the model iteratively processed the training data over multiple epochs. For each epoch, the network performed a forward pass to analyze the input data and a backward pass to propagate errors, updating the model weights. This iterative process allowed the model to uncover intricate relationships and patterns within the data, essential for accurate asthma diagnosis.

The ADENA model was further enhanced by incorporating thousands of pre-defined parameter ranges derived from prior research and clinical feedback. These inputs provided a robust foundation for model training, ensuring the inclusion of relevant clinical insights. Certain parameters, identified as more critical for asthma diagnosis, were assigned higher weights during algorithm construction to prioritize their influence on the model’s predictions.^13^ This comprehensive training process ensured that ADENA could effectively learn from diverse data inputs and deliver reliable diagnostic outcomes.

### 3.2. Evaluation metrics

Several evaluation metrics were employed to assess the performance of the ADENA framework, including mean squared error (MSE), F1 score, receiver operating characteristic (ROC) curve, area under the curve (AUC), and accuracy. The MSE was used to quantify prediction error by calculating the average squared difference between the actual labels *y_actual_* and the predicted probabilities *y_predicted_*:


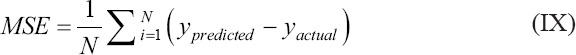


This metric provides insight into how well the model predicts outcomes on a continuous probability scale. The F1 score, a harmonic mean of precision and recall, was applied to evaluate the model’s balance between false positives (FP) and false negatives (FN). This is particularly critical in asthma diagnosis, where borderline cases can be easily misclassified. By capturing the trade-off between precision and recall, the F1 score highlights the model’s effectiveness in identifying challenging cases:


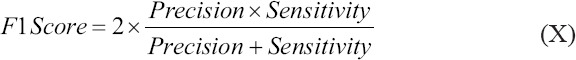


The model’s ability to discriminate between asthmatic and non-asthmatic cases was examined using the ROC curve and its corresponding AUC. These metrics illustrate the model’s performance across various threshold settings, providing a comprehensive evaluation of its discriminatory power. A high AUC indicates the model’s strong ability to distinguish between positive and negative cases. Finally, the overall correctness of the model was measured using accuracy, which calculates the proportion of correctly identified cases, including both true positives (TP) and true negatives. This metric reflects the model’s overall reliability in categorizing individuals as asthmatic or non-asthmatic.


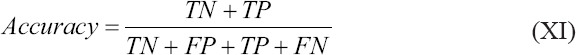


Together, these evaluation metrics offer a robust and multi-faceted assessment of ADENA’s diagnostic capabilities, demonstrating its effectiveness in accurately identifying asthma while minimizing errors.

### 3.3. Principal component analysis and deconvolution

**Figure 2:**
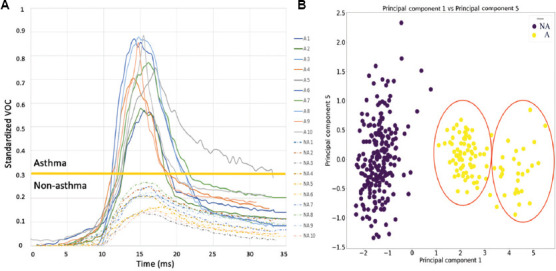
The distinctions in volatile organic compound (VOC) profiles between asthmatic and non-asthmatic individuals. (A) VOC graphs highlight differences in compound presence and intensity. (B) Principal component analysis (PCA) reveals clear separation between asthmatic (yellow) and non-asthmatic (purple) groups, with red circles indicating potential phenotypic clustering among asthmatics.

The PCA reveals significant differences between individuals with asthma (yellow) and those without asthma (purple) when specific parameters are analyzed (Figure 2A). The results highlight distinct groupings and clusters, particularly within the asthmatic group, as evidenced by the red circles in Figure 2A. These groupings reflect the potential to identify various asthma phenotypes, indicating that VOC attributes are highly informative in diagnosing and categorizing different asthma endotypes. The PCA graphs illustrate CO_2_ waveform traits on the x-axis and VOC characteristics on the y-axis, underscoring the importance of these parameters in distinguishing between asthmatic and non-asthmatic profiles. This reinforces the utility of incorporating all available parameters into the deep learning model, broadening its scope and improving its accuracy in classifying individuals based on their asthma status.

Our analysis of VOC data employed targeted metrics to provide a clear distinction between adults with and without asthma. Metrics, such as VOC plateau width and VOC maximum value play a critical role in identifying volatile compound emissions that differentiate the two groups. Figure 2B illustrates these differences, with the VOC maximum value and plateau width offering key insights into the separation of asthmatic and non-asthmatic profiles. This methodology enhances diagnostic accuracy and supports the identification of characteristic patterns in the data.

**Figure 3:**
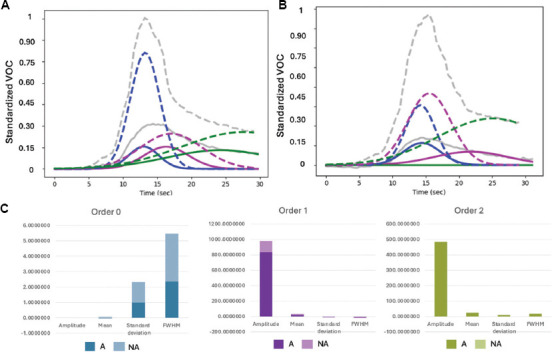
Standardized volatile organic compound (VOC) profiles deconvoluted across three retention time orders and key signal characteristics. (A and B) Standardized volatile organic compound graphs deconvoluted across three different orders to highlight differences between asthmatic (A) and non-asthmatic adults. (C) Bar charts showing the average amplitude, mean, standard deviation, and full-width half maximum across asthmatic and non-asthmatic adults.

The deconvolution analysis further emphasizes these distinctions by quantifying differences in amplitude, mean, standard deviation (SD), and full-width half maximum (FWHM) across asthmatic and non-asthmatic groups (Figure 3, Table 1). At Order 0, asthmatics exhibited a 207.14% higher amplitude and a 205.88% increase in mean compared to non-asthmatics, although the differences in SD (1.44%) and FWHM (1.69%) were minimal. At Order 1, the differences became more conspicuous, with asthmatics demonstrating dramatic increases in amplitude and mean, along with a substantial 236% rise in both SD and FWHM, indicating broader peaks and greater variability. By Order 2, asthmatics continued to show elevated values, including a 97.33% increase in amplitude and moderate increases in mean (14.14%), SD (14.72%), and FWHM (14.64%).

**Table 1:** Quantitative results of deconvolution between asthmatic (A) and non-asthmatic (NA) adults, showing average amplitude (AMP), mean, SD, and FWHM

Order/class	AMP	Mean	SD	FWHM
0				
A	0.00258	0.026	1.022	2.41
NA	0.00084	0.0085	1.0075	2.37
1				
A	327.86	17.04	4.51	10.61
NA	0.0027	0.088	1.34	3.15
2				
A	343.15	27.61	8.97	21.12
NA	173.91	24.18	7.82	18.42

Abbreviations: A: Asthmatic; NA: Non-asthmatic; AMP: Amplitude; SD: Standard deviation; FWHM: Full-width half maximum.

**Figure 4:**
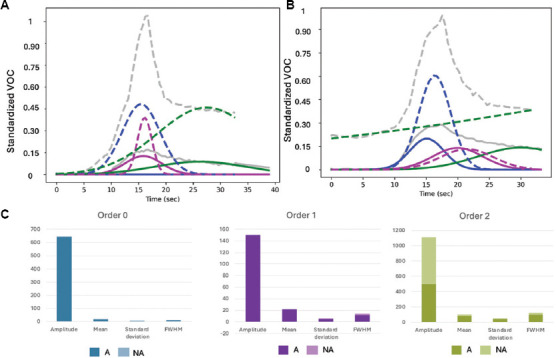
Standardized VOC profiles and statistical comparisons highlight differences in retention time and distinct signal characteristics between pediatric asthmatic and non-asthmatic individuals. (A and B) Standardized volatile organic compound graphs deconvoluted across three different orders to highlight differences between pediatric asthmatic (A) and non-asthmatic individuals. (C) Bar charts showing average amplitude, mean, SD, and FWHM across pediatric asthmatics and non-asthmatics.

Our analysis of VOC data for the pediatric group highlights clear distinctions between asthmatic and non-asthmatic profiles across key metrics, including amplitude, mean, SD, and FWHM (Figure 4, Table 2). At Order 0, asthmatic children exhibited an amplitude over 83,000 times higher and a mean 11,838% greater than non-asthmatic children. These results indicated sharper, more intense peaks in asthmatic profiles, reflecting significantly elevated volatile emissions compared to the control group. At Order 1, non-asthmatic children showed 250% higher amplitude and a mean value 323% greater than asthmatic children, whose values approach baseline. Non-asthmatics also displayed broader peaks, with SD and FWHM increasing by 80% and 136%, respectively, further differentiating the two groups. At Order 2, asthmatic children again demonstrated stronger signals, with an amplitude 23% higher, although their mean was 68% lower than non-asthmatics. However, asthmatics exhibited far greater variability, with SD increasing by 354% and FWHM by 14% compared to non-asthmatics.

**Table 2:** Quantitative results of deconvolution between pediatric asthmatics (A) and non-asthmatics (NA), showing average amplitude (AMP), mean, SD, and FWHM

Order/class	AMP	Mean	SD	FWHM
0				
A	643.18	15.52	3.51	8.26
NA	0.000774	0.13	1.31	3.17
1				
A	6.02E-10	5.08E-09	4.96	11.68
NA	150.33	21.56	0.99	2.35
3				
A	612.02	27.85	42.11	21.81
NA	497.99	16.56	9.26	19.18

Abbreviations: A: Asthmatic; NA: Non-asthmatic; AMP: Amplitude; SD: Standard deviation; FWHM: Full-width half maximum.

These findings revealed that asthmatics consistently displayed stronger signals, broader peaks, and greater variability than non-asthmatics, especially in higher-order analyses. This pattern underscores the efficacy of deconvolution and VOC analysis in enhancing the diagnostic framework, allowing for a more nuanced understanding of asthma phenotypes and facilitating improved diagnosis and management of the condition.

### 3.4. Comparative study

An extensive evaluation was conducted on the proposed model, ADENA, to determine the optimal hyperparameter settings, such as learning rates, batch size, epochs, and optimization algorithms. Through systematic experimentation, the batch size was set to four, balancing computational efficiency with accuracy. The learning rate and number of epochs were optimized to 5 × 10^−4^ and 200, respectively. These values produced the best results in a reasonable time frame, ensuring stable convergence throughout training.

**Figure 5:**
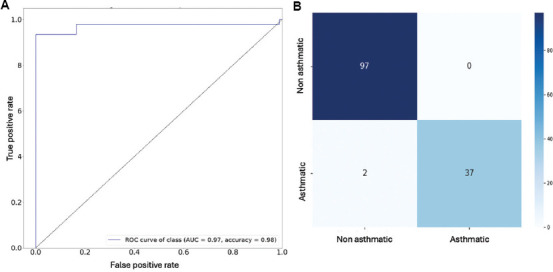
Model performance in distinguishing asthmatic from non-asthmatic cases is evaluated through diagnostic accuracy via ROC curve and classification outcomes summarized by the confusion matrix. (A) The receiver operating characteristic curve shows the model’s ability to differentiate between asthmatic and non-asthmatic cases, with area under the curve representing diagnostic accuracy. (B) Confusion matrix showing the summary of the model’s classification performance, displaying true positives, true negatives, false positives, and false negatives on the testing dataset.

The performance of the ADENA framework was compared against several deep learning architectures and traditional machine learning models, using metrics, such as accuracy, F1 score, and MSE. Table 3 shows that ADENA outperformed its counterparts in asthma diagnosis. For instance, the SVM, a popular traditional machine learning model, struggled to provide consistent performance. It achieved 74.5% accuracy, an F1 score of 0.38, and a relatively high MSE of 2.55, indicating poor generalization and difficulty in handling the data’s complex, non-linear relationships. In contrast, ADENA yielded outstanding results, with an accuracy of 98.7%, an F1 score of 0.98, and a remarkably low MSE of 0.065, demonstrating its precision and dependability in distinguishing asthmatic from non-asthmatic profiles. This performance is attributed to ADENA’s advanced feature extraction and attention mechanisms, which enable it to detect subtle patterns in input data. Residual network (ResNet) outperformed other deep learning models, with an accuracy of 97.1% and an F1 score of 0.95. However, its MSE of 0.21 remained significantly higher than that of ADENA, indicating that, while ResNet performs well, it lacks the precision and low error rates achieved by ADENA’s architecture. Similarly, the multilayer perceptron (MLP) and classification and regression tree (CART) models had similar F1 scores of 0.94 and 0.93, with accuracies of 93.8% and 94.7%, respectively. However, their higher MSE values (0.29 for MLP and 0.14 for CART) demonstrated ADENA’s ability to generalize more effectively and reduce prediction errors. The Naive Bayes (NB) classifier performed reasonably well, with an accuracy of 91.6% and an F1 score of 0.89. Nonetheless, its higher MSE of 0.58 suggests that NB struggles with the complexity and non-linearity inherent in asthma diagnosis, restricting its ability to produce consistently reliable results. To evaluate the model’s ability to distinguish between TPs and FPs, the AUC for the ROC curve was calculated (Figure 5). ADENA attained an AUC of 0.98, demonstrating its high discriminatory power and ability to accurately classify cases across a wide range of thresholds. These findings emphasize that ADENA’s architecture, which focuses on identifying subtle and complex patterns in data, is critical to achieving cutting-edge performance.

**Table 3:** Quantitative results of different models compared to the proposed method in terms of average accuracy, F1 score, and MSE

Model	Accuracy (%)	F1 score	MSE
SVM^16^	74.5	0.38	2.55
KNN^17^	95.6	0.93	0.43
LR^18^	82.2	0.53	0.64
ResNet^19^	97.1	0.95	0.21
MLP^20^	93.8	0.94	0.29
CART^21^	94.7	0.93	0.14
NB^22^	91.6	0.89	0.58
ADENA	98.7	0.98	0.065

Abbreviations: ADENA: Asthma diagnostic enhanced neural architecture; CART: Classification and regression tree; KNN: K-nearest neighbors; LR: Logistic regression; MLP: Multilayer perceptron; NB: Naive Bayes; ResNet: Residual network; SVM: Support vector machine; MSE: Mean squared error.

## 4. Discussion

The ADENA model outperformed traditional machine learning approaches and cutting-edge deep learning models in asthma diagnosis. To accomplish this, hyperparameters, such as batch size, learning rate, and number of epochs were carefully tuned. These settings enabled ADENA to deliver outstanding computational efficiency while producing highly accurate results. Notably, ADENA achieved an impressive accuracy of 98.7%, outperforming traditional models, such as SVM, which achieved only 74.5%. This significant improvement demonstrates ADENA’s reliability and precision in accurately identifying asthma profiles, making it an effective tool for clinical use.

ADENA’s F1 score of 0.98, which mirrors a well-balanced model that can reduce FPs and FNs, further supports its exceptional performance. This stands in stark contrast to the SVM’s F1 score of 0.38, highlighting the limitations of conventional approaches in managing complex diagnostic tasks. Furthermore, the model outperformed competing deep learning models, such as ResNet (MSE = 0.21) and MLP (MSE = 0.29), achieving an exceptionally low MSE of 0.065. These outcomes demonstrate ADENA’s strong generalization across datasets, producing accurate predictions even in the presence of non-linear data complexities. While deep learning models, such as ResNet performed impressively, with an accuracy of 97.1% and an F1 score of 0.95, ADENA’s sophisticated architecture, which included feature extraction methods and attention mechanisms, allowed it to attain lower error rates and superior overall performance. Similarly, models, such as MLP and CART yielded respectable accuracy rates of 94.7% and 93.8%, respectively. However, their higher MSE values suggest that these models are less effective in identifying the nuanced, non-linear correlations present in asthma-related data, something that ADENA captures more accurately.

The deconvolution analysis showed distinct physiological differences between asthmatic and non-asthmatic patients across all measured parameters, offering a more comprehensive explanation for ADENA’s efficacy. At Order 0, asthmatics exhibited mean values 205% higher and amplitudes 207% greater than non-asthmatics, although the differences in FWHM (1.69%) and SD (1.44%) were negligible. These disparities were particularly pronounced at Order 1, where asthmatics showed notable increases in mean and amplitude, as well as a 236% rise in both FWHM and SD. This indicates broader peaks and more fluctuations in asthmatic profiles. At Order 2, asthmatics continued to display elevated values, with amplitude increasing by 97% and moderate increases in mean, SD, and FWHM. These results suggest stronger signals and wider peaks in higher-order analyses. However, a limitation of this study is the exclusion of patients with other obstructive airway diseases, such as chronic obstructive pulmonary disease (COPD). Given that capnographic abnormalities can appear in various forms of airway obstruction, future studies must include such groups to further evaluate the specificity of our model. In addition, we acknowledge that Exhale-Dx does not collect FeNO, which limits the present system’s utility, particularly in asthma phenotyping. However, the modular nature of the platform allows for the potential integration of FeNO sensing technology in future iterations, which could enhance its clinical utility in distinguishing T2-high from T2-low asthma endotypes.

This deconvolution analysis is critical for understanding the physiological patterns detected by ADENA’s feature extraction techniques. The significant variations in peak width, variability, and amplitude between the asthmatic and non-asthmatic groups underscore ADENA’s ability to capture subtle patterns that conventional models, such as SVM and even ResNet might overlook. These outcomes confirm the model’s potential to identify and classify different asthma phenotypes effectively. With an AUC of 0.98 in the ROC analysis, ADENA demonstrated strong discriminatory power in distinguishing TPs from FPs, further reinforcing its clinical value. Even with small datasets, ADENA’s high accuracy and low error rates indicate its promise for practical integration into healthcare systems, where accurate and reliable asthma diagnosis is essential.

## 5. Conclusion

The ADENA model has proven to be a highly effective and reliable tool for asthma diagnosis, outperforming both traditional machine learning and modern deep learning models. By combining optimized hyperparameters, advanced attention mechanisms, and feature extraction techniques, ADENA achieved exceptional performance, delivering an accuracy of 98.7%, an F1 score of 0.98, and a low MSE of 0.065. The deconvolution analysis further confirmed the model’s ability to detect significant physiological differences between asthmatic and non-asthmatic profiles, particularly in terms of amplitude, variability, and peak characteristics across multiple orders. These findings demonstrate ADENA’s capability to capture complex, non-linear patterns, providing a reliable solution for non-invasive asthma diagnostics and phenotypic identification.

Future efforts will be directed at expanding the dataset to include a broader range of patient demographics, phenotypes, and clinical scenarios, thereby improving the model’s generalizability and robustness. ADENA will also be expanded to classify other respiratory diseases, such as COPD and bronchitis, further enhancing its clinical utility. Large-scale, real-world validation through clinical trials will be carried out to ensure its accuracy and practical integration into healthcare workflows. Finally, efforts to improve computational efficiency will allow for real-time predictions, paving the way for ADENA to become a transformative tool in respiratory disease diagnosis.

## Data Availability

Data from this study are available from the corresponding author upon reasonable request.
